# The impact of PECS II blockade in patients undergoing minimally invasive cardiac surgery—a prospective, randomized, controlled, and triple-blinded trial

**DOI:** 10.1186/s13063-023-07530-7

**Published:** 2023-09-04

**Authors:** Elisabeth Hoerner, Ottokar Stundner, Felix Naegele, Anna Fiala, Nikolaos Bonaros, Peter Mair, Johannes Holfeld, Lukas Gasteiger

**Affiliations:** 1 Department of Anaesthesiology and Intensive Care Medicine, Medical University of Innsbruck, Anichstrasse 35, Innsbruck, 6020 Austria; 2 Department of Cardiac Surgery, Medical University of Innsbruck, Anichstrasse 35, Innsbruck, 6020 Austria

**Keywords:** PECS II block, Fascial plane block, Minimal invasive cardiac surgery, Clinical trial

## Abstract

**Background:**

Classic neuraxial techniques, such as thoracic epidural anesthesia, or alternative approaches like the paravertebral block, are not indicated in cardiac surgery due to increased bleeding risk. To provide satisfactory analgesia without the need for excessive opioid use, novel ultrasound techniques gained popularity and are of growing interest. The pectoralis nerve block II (PECS II) has been shown to provide good postoperative analgesia in modified radical mastectomy and might also be suitable for minimally invasive cardiac surgery.

**Methods:**

In a single center, prospective, triple-blinded, two-group randomized trial, 60 patients undergoing elective, unilateral minimal invasive cardiac surgery will be randomized to receive a PECS II with 30 ml of ropivacaine 0.5% (intervention group) or sodium chloride 0.9% (placebo group). The primary outcome parameter is the overall opioid demand given as intravenous morphine milligram equivalents (MME) during the first 24 h after extubation. Secondary endpoints are the visual analog scale (VAS) 2, 4, 6, 8, 12, and 24 h after extubation, the Overall Benefit of Analgesia Score (OBAS) after 24 h, the interval until extubation, and intensive care unit (ICU) discharge within 24 h, as well as the length of hospital stay (LOS).

**Discussion:**

This prospective randomized, controlled, and triple-blinded trial aims to assess if a PECS II with ropivacaine 0.5% helps to decrease the opioid demand in the first 24 h and increases postoperative pain control after minimally invasive cardiac surgery.

**Trial registration:**

www.clinicaltrialsregister.eu; EudraCT Nr: 2021–005452-11; Lukas Gasteiger MD, November 18, 2021.

## Administrative information

**Table:** 

Title {1}	The impact of PECS II blockade in patients undergoing minimal invasive cardiac surgery– A prospective, randomized, controlled and triple-blinded trial
Trial registration {2a and 2b}	www.clinicaltrialsregister.eu; EudraCT Nr: 2021–005452-11; Lukas Gasteiger MD, November 18 2021
Protocol version {3}	Rev. No. 1.7, Date: 21.04.2022
Funding {4}	The PECS-MICS trial is investigator-initiated at the Medical University of Innsbruck
Author details {5a}	Elisabeth Hoerner Department of Anaesthesiology and Intensive Care Medicine, Medical University of Innsbruck, Anichstrasse 35, 6020 Austria Ottokar Stundner Department of Anaesthesiology and Intensive Care Medicine, Medical University of Innsbruck, Anichstrasse 35, 6020 Austria Felix Naegele (corresponding author) Department of Cardiac Surgery, Medical University of Innsbruck, Anichstrasse 35, 6020 Austria Anna Fiala Department of Anaesthesiology and Intensive Care Medicine, Medical University of Innsbruck, Anichstrasse 35, 6020 Austria Nikolaos Bonaros Department of Cardiac Surgery, Medical University of Innsbruck, Anichstrasse 35, 6020 Austria Johannes Holfeld Department of Cardiac Surgery, Medical University of Innsbruck, Anichstrasse 35, 6020 Austria Peter Mair Department of Anaesthesiology and Intensive Care Medicine, Medical University of Innsbruck, Anichstrasse 35, 6020 Austria Lukas Gasteiger Department of Anaesthesiology and Intensive Care Medicine, Medical University of Innsbruck, Anichstrasse 35, 6020 Austria
Name and contact information for the trial sponsor {5b}	Medical University of Innsbruck Innrain 52, Christoph-Probst-Platz 1, 6020 Innsbruck, Austria Contact: mui-sponsor@i-med.ac
Role of sponsor {5c}	"The sponsor did not provided funding for this study and played no part in the study design, data collection, data management, data analysis, data interpretation, report writing, or the decision to submit the report for publication. The study was conducted independently by the authors."

## Introduction

### Background and rationale {6a}

A multidisciplinary approach in perioperative care is crucial for improving patient recovery after major surgery. The so-called Enhanced Recovery After Surgery (ERAS) is an evidence-based protocol ranging from preoperative patient optimization to intra- and postoperative strategies aiming to improve clinical outcomes and reduce hospital stays. With the introduction of minimally invasive cardiac surgery (MICS) through two or three small thoracotomies, ERAS strategies gained popularity, and recently, ERAS guidelines for cardiac surgery have been presented, emphasizing that critical factors that keep patients hospitalized after major cardiac surgery are among others, perioperative pain, prolonged mechanical ventilation, and immobilization [1–3].

Apart from less invasive surgical techniques, implementing a multimodal concept for pain therapy, including regional anesthesia, is an essential element of ERAS pathways [4]. In patients undergoing major cardiac surgery, the possibilities for neuraxial blocks are limited and controversially discussed due to increased bleeding risk [5].

Ultrasound-guided, interfacial plane blocks represent a relatively new route of transmitting local anesthetics to the space between two fascial layers to provide the blockade of nerves traveling within this plane, leading to analgesia of the chest or abdominal wall. These blocks have already been shown to be relatively easy to perform and to have low complication risks [5, 6].

The pectoral nerves (PECS) II block was initially introduced in 2012 as an extension of the PECS I block (injection of local anesthetic between the pectoralis major and pectoralis minor muscles), involving a second injection in the fascial plane between pectoralis minor and serratus anterior muscles [7, 8]. It targets the pectoral nerves, the intercostal nerves III-VI, the intercostobrachial, and the long thoracic nerve. Compared to systemic opioids, the PECS II block has already been proven to effectively facilitate pain relief to the upper anterior chest wall in patients undergoing modified radical mastectomy [9–11]. Looking at the unilateral right-sided approach of MICS, a PECS II block could be a suitable regional anesthetic technique to improve postoperative analgesia for patients undergoing MICS [5].

Therefore, this prospective, triple-blinded, randomized trial aims to evaluate the efficacy of the PECS II block in patients undergoing unilateral MICS.

### Objectives {7}

The primary objective of this trial is to evaluate the efficacy of the PECS II block in patients undergoing unilateral MICS expressed as the reduction in the overall opioid demand given as intravenous MME during the first 24 h after extubation.

The secondary objective of this trial is to evaluate the effect of the PECS II block in patients undergoing unilateral MICS on the interval until extubation, ICU discharge, data on ICU, length of hospital stay (LOS), the visual analog scale (VAS) at 2, 4, 6, 12, and 24 h after extubation, and the Overall Benefit of Analgesia Score (OBAS) at 24 h after extubation [12].

### Trial design {8}

This is an investigator-initiated single-center prospective, triple-blinded, two-group, randomized trial with two treatment groups.

## Methods: participants, interventions, and outcomes

### Study setting {9}

**Fig. 1:**
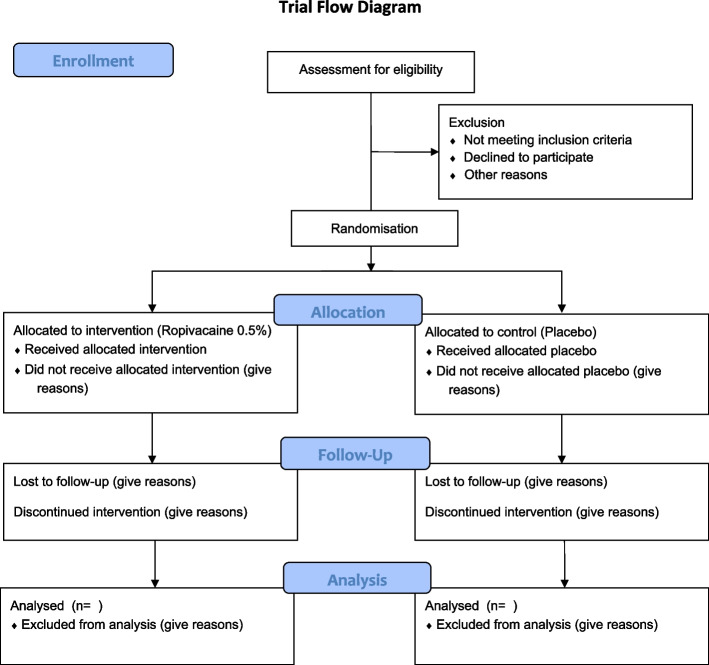
Trial flow diagram

In this single-center prospective, triple-blinded, two-group, randomized trial, 60 patients will be recruited and randomly assigned to one of the two groups to test the effect of a PECS II block with ropivacaine 0.5% on the postoperative opioid demand (see Consolidated Standards of Reporting Trials flow diagram (CONSORT) [13] in Fig. 1). The scope of this trial is to improve perioperative pain management in patients undergoing minimally invasive cardiac surgery.

The primary outcome parameter is the overall opioid demand given as intravenous MME during the first 24 h after extubation.

The secondary outcome variables are the interval until extubation, ICU discharge, data on ICU, length of hospital stay (LOS), the VAS at 2, 4, 6, 12, and 24 h after extubation, and the Overall Benefit of Analgesia Score (OBAS) at 24 h after extubation [12].

This study will be performed in the Innsbruck Medical University Hospital. All participants will be recruited by a medical specialist team (recruiting team) at the Medical University of Innsbruck (MUI) (Investigation Site). All data will be collected by the research team consisting of physicians and healthcare researchers. The study investigators will perform data analysis.

### Eligibility criteria {10}

Adult patient with an American Society of Anesthesiologist (ASA) physical status I-III, aged 18–80 years, scheduled for elective minimal invasive cardiac surgery, e.g., mitral valve procedures, tricuspid valve procedures, myxoma resection, or atrial septal defect repair. All participants will undergo the usual standard medical work-up before, during, and after the procedure according to the ESC/EACTS Guidelines on Valvular Heart Disease [14].

This includes echocardiography, spirometry, carotid artery duplex scan, cardiac MRI, and/or computed tomography, and interviews with the cardiologist, cardiac surgeon, and anesthesiologist.

To be eligible for this study, patients must meet all of the following criteria:

- Age: 18–80 years.
- BMI 18–35 kg/m.^2^
- Informed consent.
- MICS

The exclusion criteria are as follows:

- Known allergies to administered drugs.
- Severe diabetic neuropathy.
- LV-dysfunction (EF < 30%).
- Drug-opioid abuse.
- Patients with chronic pain syndrome.
- Systemic infection.
- Psychiatric disorders that lead to alteration in the perception and evaluation of pain.
- Pregnancy.
- Anticipated endotracheal intubation > 24 h.
- eGFR < 30 or chronic renal disease.

### Who will take informed consent? {26a}

Every participant must give written consent before participating in the clinical trial.

An investigator will provide and obtain the informed consent form. Therein, the investigator will thoroughly inform the participant in oral and written form in an understandable manner for the patient about the character, importance, relevance, and consequences of the clinical trial. Before the participant may sign the informed consent form, there will be ample opportunity to consider participation and discuss questions with the investigators. The informed consent file must be signed and dated by the participant and the investigator and stored in the investigator site file. Nevertheless, if the participant decides not to participate any time before the intervention, he will not be included without negative impacts on the procedure.

### Additional consent provisions for collection and use of participant data and biological specimens {26b}

On the consent form, participants will be asked if they agree to the use of their data should they choose to withdraw from the trial. This includes their data being used for analysis as well as any future publications or presentations related to the study. Since this is a monocenter trial, the consent form will not include a section regarding data sharing permissions. This trial does not involve collecting biological specimens for storage. All necessary measures will be taken to protect the privacy and confidentiality of participants. Participants will also be provided with the contact information for the study team in case they have any questions or concerns regarding their participation in the study.

### The explanation for the choice of comparators {6b}

Two groups will be included. The intervention group receives PCS II block with 30 ml of ropivacaine 5 mg/ml, while the control group receives a plane PECS II block with 30 ml of sodium chloride 0.9%. All other medical interventions will be identical.

### Intervention description {11a}

After obtaining written informed consent, all patients will be taught to assess pain using the visual analog scale (VAS; 0–100 mm). According to institutional standards, patients will be fasted for 6 h for solids and 2 h for fluids and receive oral premedication with midazolam 0.05–0.1 mg /kg if requested.

Before the arrival of the patient in the operating room, a study nurse will open an opaque envelope and prepare the study medication (30 ml ropivacaine 0.5% for the intervention group and 30 ml sodium chloride 0.9% for the placebo group) according to the study randomization and hand it to the blinded anesthetist.

Upon arrival at the operating room, standard monitoring (5-channel electrocardiogram, pulse oximetry (SpO_2_), a peripheral vein cannula, an arterial cannula for continuous invasive blood pressure measurement, and near-infrared spectroscopy (NIRS)) will be installed. Then, all patients will be pre-oxygenated for 3 min with oxygen 100% according to international standards. Anesthesia induction will be performed with midazolam 10-–20 mcg/kg, fentanyl 5–8 mcg/kg, propofol 1–3 mg/kg, and rocuronium 1 mg/kg, and the patient airway will be secured by using an endotracheal tube. All patients will receive controlled ventilation (tidal volume 6–8 ml/predicted body weight) to maintain arterial partial pressure of carbon dioxide (pCO_2_) between 35 and 45 mmHg.

A five-lumen central venous catheter will be inserted in the right subclavian or internal jugular vein, depending on the indication of a bicaval extracorporeal venous drainage for cardiopulmonary bypass. Immediately afterward, the ultrasound-guided PECS II block will be performed under aseptic conditions on the right hemithorax using the technique described by Blanco et al. [8].

According to our institutional standards, a trans-esophageal echocardiographic probe will be inserted in all patients to control and assess cardiac function.

All PECS II blocks will be performed under ultrasound guidance by five consultant anesthetists experienced in the technique (*n* > 50) by using a 22 G × 80 mm needle (SonoPlex cannula, Pajunk®, Geisingen, Germany) and a linear array ultrasound (US) probe (FUJIFILM Sono-Site M-Turbo®, 6–13 MHz). At the midclavicular line, the axillary artery and vein are identified. The linear ultrasound probe is moved caudally and laterally to delineate the pectoralis major, pectoralis minor, and serratus muscle at the third intercostal space. Then, the needle is inserted in-plane from cranial medial to caudal lateral in an oblique manner until the tip is visualized between the pectoralis minor and serratus muscle, and 20 ml of the study medication is injected. After that, the information will be removed until it reaches the plane between the pectoralis major and pectoralis minor muscle, and the remaining 10 ml of the study medication will be injected. Heart rate (HR), blood pressure, and SpO_2_ will be noted immediately before the PECS II block is performed and 5 min after.

### Postoperative analgesia regimen

At the end of the surgical procedures, all patients will receive a bolus of piritramide 0.1–0.2 mg/kg and will be transferred to the postoperative ICU ward. Patients will be extubated when extubation criteria are reached (normothermic, fully awakened, spontaneously breathing, hemodynamically stable with moderate vasopressor support, etc.). After extubation, VAS will be recorded by trained medical staff members and then after 2, 4, 6, 8, 12, and 24 h. If VAS exceeds a value of 30 mm at any time, an administration of piritramide will be allowed. The time interval from completing the PECS II block until the first request for opioids (VAS > 30 mm) after extubation is defined as the duration of analgesia. Opioid consumption will be assessed as an intravenous morphine milligram equivalent (MME) dose.

### Criteria for discontinuing or modifying allocated interventions {11b}

We will exclude patients from participating in case of failure to detect the suitable anatomical structures for PECS II.

After PECS II is performed, it is impossible to change the allocated intervention after administration. Subjects are free to leave the study at any time for any reason without consequences. Study discontinuation should be documented in the subject’s medical file and the case report form (CRF). The investigator can withdraw an issue from the study for urgent medical reasons. These may include the following criteria:

- Anatomical anomalies, limited visualization
- Allergic reaction to the study medication
- Clinical complications (hemodynamic impairment, re-operation, extracorporeal membrane oxygenation (ECMO), etc.) that may lead to prolonged weaning (more than 24 h)

### Strategies to improve adherence to interventions {11c}

To improve adherence to the study protocol, the follow-up visits for the study are planned simultaneously with the standard measurements for patients in the MICS program at the Medical University of Innsbruck. Participants do not need to adhere to specific tasks. To support faculty staff, a study nurse who reviews the patient files throughout the study period will be involved.

### Relevant concomitant care permitted or prohibited during the trial {11d}

There is no expected interaction between the drug under investigation and concomitant medical treatments. Local prescribing information and institutional guidelines should be followed as applicable.

### Provisions for post-trial care {30}

The sponsor has insurance by the legal requirements in Austria. This insurance covers damage to research participants through injury or death caused by the study. The insurance applies to the damage that becomes apparent during the study.

### Outcomes {12}

The primary outcome parameter is the overall opioid demand given as intravenous MME during the first 24 h after extubation.

Secondary outcome variables are the interval until extubation, ICU discharge, data collected in the ICU, length of hospital stay, the VAS at 2, 4, 6, 12, and 24 h after extubation, and the Overall Benefit of Analgesia Score (OBAS) at 24 h after extubation [12].

### Participant timeline {13}

Demographic and baseline data such as age, weight, height, and ASA score are collected soon after informed consent is granted, usually the day before surgery. On the day of surgery, data that define the type of surgery and the type and size of central venous cannulation will be noted. Also, hemodynamic data such as arterial pressure, heart frequency, and blood oxygen saturation before and soon after the performance of the PECS block will be recorded. At the end of the surgery, the amount of opioids and sedatives used during the surgery will be noted. The time of ICU admission and hemodynamic data will be noted at ICU admission.

All opioids and other analgesics will be noted until the extubation and after 2, 4, 6, 8, 12, and 24 h. Also, the NRS score will be assessed at the defined time points.

After 24 h, the OBAS assessment will be performed, and the study participation will end.

### Sample size {14}

The main hypothesis of this study is that the use of PECS II blockade in patients undergoing minimally invasive cardiac surgery reduces the overall opioid demand given as morphine milligram equivalents (MME) during the first 24 h after extubation. The sample size needed to test this hypothesis was based on four previous studies [15–18]. Study results were pooled and weighted due to considerable heterogeneity in variances. Assuming an average weighted effect size of 0.75 (Cohen’s *d*) aiming for a statistical power of 80% and a testing significance level of 5%, a sample size of 28 patients per group is required. Considering a potential dropout rate of 7%, we enrolled 60 patients (30 patients per group).

### Recruitment {15}

The MICS program at the University Clinic of Cardiac Surgery, Medical University of Innsbruck, serves as the primary recruitment institution.

## Assignment of interventions: allocation

### Sequence generation {16a}

The investigational medication (ropivacaine 0.5%) assigned to individual patients is determined by a randomized schedule with a 1:1 allocation ratio.

### Concealment mechanism {16b}

The randomization process will be realized independently from the clinical investigators using opaque envelopes (fully blind randomization). For each participant enrolled in the study, one envelope will be opened by the nurse who prepares the study medication. Preparation will be performed in a separate room without the attendance of any study investigator.

### Implementation {16c}

The randomization list will be generated using well-established software (randomization.org) for the two groups (intervention group, placebo group). Upon the arrival of the patient in the operating theatre, a study nurse will open an opaque envelope which is labeled with the patient’s identification code and prepare the study medication (30 ml ropivacaine 0.5% for the intervention group and 30 ml sodium chloride 0.9% for the placebo group) according to the study randomization and hand it to the blinded anesthetist.

## Assignment of interventions: blinding

### Who will be blinded {17a}

This study is, per definition, triple-blinded as the participants, care providers, and investigators involved in the study procedure do not know the randomization arm. All study assessments will be performed in an observer-blinded fashion.

### Procedure for unblinding if needed {17b}

A patient identification code will replace patient identifying data. The randomization list and the codebook will be stored digitally and only accessible to study personnel. Unblinding is possible at all times after consultation with the principal investigator.

## Data collection and management

### Plans for assessment and collection of outcomes {18a}

All investigators will be trained within the initiation visit. Training topics cover Good Clinical Practice, the study protocol, and OBAS and VAS assessment execution.

The patient’s demographical data will be registered soon after the patient’s informed consent. The PECS II block will be performed by experienced anesthesiologists who have completed at least 50 procedures. All data will be collected using a paper-based CRF. The healthcare data of participants will be derived from the electronic patient file. All data will be stored for 15 years, and results will be published in scientific journals.

### Plans to promote participant retention and complete follow-up {18b}

After enrollment and randomization, the investigators will make every reasonable effort to follow the study subject throughout the entire study period. Subjects are free to discontinue their study participation at any time. Participant retention will be increased by implementing study visits into routine clinical postoperative care.

### Data management {19}

An independent internal monitor (Tirol Kliniken) will provide the monitoring and quality assurance of the study. The study team will process data entry from paper based CRF into statistical software. Programmed checks make data validation of range, validity, and consistency. If necessary, queries are made by the study software or an authorized person. Based on the questions, the investigator can check and clarify discrepancies.

After recording all entries and clarifying all queries, the database will be closed after the study. This performance must be documented. All data will be handled confidentially, and research data will be coded using a unique patient identification number. To reproduce the study findings and help future users understand and reuse the data, all changes made to the raw data and all steps taken in the analysis will be documented. The database files will be kept for 15 years after the study has ended.

### Confidentiality {27}

All local legal requirements regarding data protection will be adhered to. All study findings and documents will be regarded as confidential. The Investigator and research team members must not disclose any information without prior written approval from the Sponsor.

The pseudonymity of patients participating must be maintained. The patients will be recognized on CRFs and other documents by age and identification number throughout documentation and evaluation. Records that identify the patient personally (e.g., the signed informed consent) must be maintained in confidence by the investigator. The patients will be told that all study findings will be stored on a computer and handled in the strictest confidence.

### Plans for collection, laboratory evaluation, and storage of biological specimens for genetic or molecular analysis in this trial/future use {33}

Not applicable; no biological specimens will be collected for this study.

## Statistical methods

### Statistical methods for primary and secondary outcomes {20a}

Data will be entered into electronic case record files (eCRF; RedCap®, Nashville, TN). R 4.2 (The R Project for Statistical Computing, Vienna, Austria) will be used for statistical analysis. The normality of data will be assessed using graphical and inferential tests. Median/interquartile range or mean/standard deviation will be used to summarize and evaluate continuous data and count/percent for categorical data. Student’s *t*-test and *χ*2 or non-parametric equivalents will be used for intergroup distribution comparisons at defined time points. For repeated measures, analysis of variance (rmANOVA) and generalized linear models (generalized estimating equation (GEE)) will be used. Linear and/or logistic regression will be used to estimate intervention effects on primary and/or secondary outcomes, controlling for common confounding factors and effect modifiers.

### Interim analyses {21b}

This study can be classified as low risk; no interim analyses will be conducted during this trial.

### Methods for additional analyses (e.g., subgroup analyses) {20b}

No subgroup analysis is planned, justified by the sample size and the pragmatic study design.

We anticipated that there would be meaningful differences in treatment effects across subgroups of participants.

### Sample size

Our study was designed to detect an effect of the PECS II block on 24 h postoperative opioid demand. As patients undergoing minimal invasive mitral valve surgery tend to be quite a homogenous population, to our understanding, conducting subgroup analyses would not have added much value to our study. Also, conducting subgroup analyses would have reduced our statistical power. Therefore, we were concerned that subgroup analyses would not yield meaningful results.

### Methods in analysis to handle protocol non-adherence and any statistical forms to handle missing data {20c}

#### Per-protocol population

The per-protocol-population will include all patients who received intervention or sham intervention (placebo) and surgery without major protocol deviations. Significant protocol deviations or complications (hemodynamic instability, prolonged intubation, reintubation, bleeding, reoperation) and subsequent failure to obtain primary and secondary outcome data will be documented before the database lock.

#### Intention-to-treat population

The intention-to-treat population will consist of all patients who were enrolled in the study and gave informed consent, but data is missing due to the abovementioned factors. This population will be analyzed in a separate ITT cohort. In case of withdrawals and missing data, the last observation carried forward (LOCF) procedure will be applied where appropriate.

### Plans to give access to the complete protocol, participant-level data, and statistical code {31c}

The entire protocol of the study will be published together with a manuscript on the clinical trial results. The datasets analyzed during the current study and the statistical code will be available from the corresponding author upon reasonable request. It will only be accessible to personnel directly involved in the study.

## Oversight and monitoring

### Composition of the coordinating center and trial steering committee {5d}

This is an investigator-initiated, single-center trial, run by a small study team that can be seen as the trial steering committee. Organizational support will be provided by the Competence Centre for Clinical Trials, Medical University of Innsbruck (KKS). The study team meets on a daily basis, to review routine clinical cases. Study-related topics will be discussed during this daily meeting as appropriate. The KKS will provide consultancy during the study initiation and closure phases, as well as on demand.

### Composition of the data monitoring committee, its role and reporting structure {21a}

An independent local monitor (Tirol Kliniken) will check trial quality repeatedly (at least every 6 months) and check at least 10% of the signed informed consent files (ICF). Complete data of the first five participants will be reviewed, including inclusion and exclusion criteria. The study has a low risk; therefore, there is no need for data safety monitoring board.

### Adverse event reporting and harms {22}

Although serious adverse events are not to be expected, the sponsor and the principal investigator will suspend the study if there is reasonable doubt that the continuation of the survey will compromise the safety and well-being of participants. In this case, the local ethics committee will be informed immediately, and the study will remain suspended pending a favorable decision from the local ethics committee.

Adverse events will be noted, and serious adverse events will also be reported to the local ethics committee.

### Frequency and plans for auditing trial conduct {23}

For quality assurance, monitoring audits will be performed. An independent local monitor will perform the monitoring.

Monitoring visits will be conducted to review study plan compliance, compare CRFs and individual patients’ medical records, perform an accounting of study material, and ensure that the study is conducted according to applicable regulatory requirements. CRF entries will be verified with source documentation. Monitoring visits will be performed repeatedly for at least all 6 months and check at least 10% of the signed ICFs.

### Plans for communicating significant protocol amendments to relevant parties (e.g., trial participants, ethical committees) {25}

After the protocol has been submitted to the ethics committee (EC), any substantial change will require a formal amendment. The amendment must be signed by all of the signatories to the original protocol. Once the study has started, amendments should be made only in exceptional cases. The ethics committees must be informed of all amendments. Approval must be sought for ethical aspects and obtained from the competent authorities*.*

### Dissemination plans {31a}

For all publications, the data protection of the subjects will be maintained. This includes a presentation at national and international conferences and publications in scientific journals. The study data are the property of the Medical University of Innsbruck. The data from the whole trial can be published separately. There are no publication restrictions, and the trial results will be accessible to the public.

## Discussion

This study will assess the opioid demand during the first 24 h after extubation following MICS. The available evidence on using local anesthetics in major cardiac surgery is scarce [19].

The secondary endpoints assess the evolution of VAS during the first 24 h, namely 2, 4, 6, 8, 12, and 24 h after extubation. Also, the OBAS after 24 h will be assessed, giving a more subjective evaluation of the analgesia and its subjective benefit [12]. Additional secondary endpoints will be the LOS, the time until extubation, and collected monitoring data.

Acute postoperative pain and the development of chronic pain are significant issues in cardiac surgery [5, 20, 21]. Poor postoperative pain management can lead to delayed ventilatory weaning, prolonged ICU stay, and prolonged LOS [1, 22]. For decades, opioid-based “balanced cardiac anesthesia techniques” were used to provide good analgesia and suppress sympathetic response during cardiac surgery. Apart from “classical” opioid-related side effects such as nausea, vomiting, sedation, and respiratory depression, the role of opioid-based anesthesia in developing the current opioid crisis in the USA is discussed [5].

With the upcoming less invasive surgical approaches, such as MICS, where two or three small thoracotomies replace open surgery access, an increased interest in ERAS pathways arose in cardiac surgery [3]. A multimodal analgesia that includes different concepts, such as regional anesthesia, can decrease opioid use and opioid-related side effects and may help reduce the length of ICU stay and LOS. Given the need for full heparinization, using neuraxial techniques or the paravertebral block (PVB) remains controversial.

Over the last decade, several ultrasound-guided truncal fascial plane blocks have been latter block has shown to provide good analgesia to the anterolateral chest wall for modified mastectomy, including axilla dissection and pectoral muscle resection [10, 18]. It is unsurprising that this block has also been used as a rescue maneuver for severe postoperative pain in cardiac surgery. It also seems to be a valuable tool for analgesia in the insertion of implantable cardioverter defibrillators (ICDs) [5, 23, 24].

Given the strict unilateral right approach of MICS, the PECS II block could be an exciting addition to multimodal analgesia. And as central venous catheters are usually placed on the right side, the ultrasound-guided PECS II block does not require further positioning, washing, and covering of the patient, thereby adding an essential advantage in time and logistics compared to other regional anesthesia techniques.

This study was designed to clarify if a PECS II block could efficiently reduce opioids in patients undergoing unilateral MICS. This study’s results may support anesthesiologists in choosing strategies for multimodal analgesia.

## Trial status

Protocol version 1.4, effective date: 21 January 2022. First patient first visit (FPFV): 08 February 2022. Currently, 36 of 50 participants are included, date: 11 October 2022. Last patient last visit (LPLV) is expected in April 2023.

## Data Availability

The datasets analyzed during the current study are available from the principal investigator or the corresponding author on reasonable request and are otherwise only accessible to the study collaborators.

## Abbreviations

ASA: American Society of Anesthesiology
CRF: Case report form
EC: Ethics committee
ECMO: Extracorporeal membrane oxygenation
ERAS: Enhanced Recovery After Surgery
FPFV: First patient first visit
GEE: Generalized estimating equation
HR: Heart rate
ICD: Implantable cardioverter defibrillator
ICF: Informed consent file
ICU: Intensive care unit
LPLV: Last patient last visit
LOS: Length of hospital stay
MICS: Minimally invasive cardiac surgery
MME: Morphine milligram equivalents
MUI: Medical University of Innsbruck
NIRS: Near-infrared spectroscopy
OBAS: Overall Benefit of Analgesia Score
PECS II: The pectoralis nerve block II
PVB: Paravertebral block
pCO2: Partial pressure of carbon dioxide
SpO2: Pulse oximetry
US: Ultrasound
VAS: Visual analog scale

## References

[1] White A, Patvardhan C, Falter F (2021). Anesthesia for minimally invasive cardiac surgery. J Thorac Dis..

[2] Engelman DT (2019). Guidelines for perioperative care in cardiac surgery: enhanced recovery after surgery society recommendations. JAMA Surg..

[3] Noss C (2018). Enhanced recovery for cardiac surgery. J Cardiothorac Vasc Anesth.

[4] Tan M, Law LSC, Gan TJ (2015). Optimizing pain management to facilitate Enhanced Recovery After Surgery pathways. Can J Anaesth.

[5] Kelava M, Alfirevic A, Bustamante S, Hargrave J, Marciniak D (2020). Regional anesthesia in cardiac surgery: an overview of fascial plane chest wall blocks. Anesth Analg.

[6] Jack JM, McLellan E, Versyck B, Englesakis MF, Chin KJ (2020). The role of serratus anterior plane and pectoral nerves blocks in cardiac surgery, thoracic surgery and trauma: a qualitative systematic review. Anaesthesia.

[7] Blanco R (2011). The ‘pecs block’: a novel technique for providing analgesia after breast surgery. Anaesthesia.

[8] Blanco R, Fajardo M, Parras MT (2012). Ultrasound description of Pecs II (modified Pecs I): a novel approach to breast surgery. Rev Esp Anestesiol Reanim.

[9] Versyck B, van Geffen GJ, Chin KJ (2019). Analgesic efficacy of the Pecs II block: a systematic review and meta-analysis. Anaesthesia.

[10] Kulhari S, Bharti N, Bala I, Arora S, Singh G (2016). Efficacy of pectoral nerve block versus thoracic paravertebral block for postoperative analgesia after radical mastectomy: a randomized controlled trial. Br J Anaesth.

[11] Hoerner E (2022). The impact of dexamethasone as a perineural additive to ropivacaine for PECS II blockade in patients undergoing unilateral radical mastectomy - a prospective, randomized, controlled and double-blinded trial. J Clin Anesth..

[12] Lehmann N (2010). Development and longitudinal validation of the overall benefit of analgesia score: a simple multi-dimensional quality assessment instrument. Br J Anaesth.

[13] Schulz KF, Altman DG, Moher D (2010). CONSORT 2010 statement: updated guidelines for reporting parallel group randomised trials. BMJ.

[14] Vahanian A (2022). 2021 ESC/EACTS guidelines for the management of valvular heart disease. Eur Heart J.

[15] Hassn AMA, Zanfaly HE, Biomy TA (2016). Pre-emptive analgesia of ultrasound-guided pectoral nerve block II with dexmedetomidine–bupivacaine for controlling chronic pain after modified radical mastectomy. Res Opin Anesth Intensive Care.

[16] Kim DH (2018). Efficacy of pectoral nerve block type II for breast-conserving surgery and sentinel lymph node biopsy: a prospective randomized controlled study. Pain Res. Manag..

[17] Wang K (2018). The efficacy of ultrasound-guided type II pectoral nerve blocks in perioperative pain management for immediate reconstruction after modified radical mastectomy: a prospective, randomized study. Clin J Pain.

[18] Versyck B, van Geffen GJ, Van Houwe P (2017). Prospective double blind randomized placebo-controlled clinical trial of the pectoral nerves (Pecs) block type II. J Clin Anesth..

[19] No Title. https://www.longdom.org/open-access/ultrasound-guided-pecs-ii-block-in-minimally-invasive-coronary-arterybypass-grafting.pdf.

[20] Gottschalk A, Cohen SP, Yang S, Ochroch EA (2006). Preventing and treating pain after thoracic surgery. Anesthesiology.

[21] Perttunen K, Tasmuth T, Kalso E (1999). Chronic pain after thoracic surgery: a follow-up study. Acta Anaesthesiol Scand.

[22] White PF (2007). The role of the anesthesiologist in fast-track surgery: from multimodal analgesia to perioperative medical care. Anesth Analg.

[23] Yalamuri S (2017). Pectoral fascial (PECS) I and II blocks as rescue analgesia in a patient undergoing minimally invasive cardiac surgery. Reg Anesth Pain Med.

[24] Pai BHP, Shariat AN, Bhatt HV (2019). PECS block for an ICD implantation in the super obese patient. J. Clin. Anesth..

